# Whole Gene Capture Analysis of 15 CRC Susceptibility Genes in Suspected Lynch Syndrome Patients

**DOI:** 10.1371/journal.pone.0157381

**Published:** 2016-06-14

**Authors:** Anne M. L. Jansen, Marije A. Geilenkirchen, Tom van Wezel, Shantie C. Jagmohan-Changur, Dina Ruano, Heleen M. van der Klift, Brendy E. W. M. van den Akker, Jeroen F. J. Laros, Michiel van Galen, Anja Wagner, Tom G. W. Letteboer, Encarna B. Gómez-García, Carli M. J. Tops, Hans F. Vasen, Peter Devilee, Frederik J. Hes, Hans Morreau, Juul T. Wijnen

**Affiliations:** 1 Department of Human Genetics, Leiden University Medical Centre, Leiden, The Netherlands; 2 Department of Pathology, Leiden University Medical Centre, Leiden, The Netherlands; 3 Department of Clinical Genetics, Erasmus University Medical Centre, Rotterdam, The Netherlands; 4 Department of Medical Genetics, University Medical Centre Utrecht, Utrecht, The Netherlands; 5 Department of Clinical Genetics, University Hospital Maastricht, Maastricht, The Netherlands; 6 Department of Clinical Genetics, Leiden University Medical Centre, Leiden, The Netherlands; 7 Department of Gastroenterology and Hepatology, Leiden University Medical Centre, Leiden, The Netherlands; Sapporo Medical University, JAPAN

## Abstract

**Background and Aims:**

Lynch Syndrome (LS) is caused by pathogenic germline variants in one of the mismatch repair (MMR) genes. However, up to 60% of MMR-deficient colorectal cancer cases are categorized as suspected Lynch Syndrome (sLS) because no pathogenic MMR germline variant can be identified, which leads to difficulties in clinical management. We therefore analyzed the genomic regions of 15 CRC susceptibility genes in leukocyte DNA of 34 unrelated sLS patients and 11 patients with *MLH1* hypermethylated tumors with a clear family history.

**Methods:**

Using targeted next-generation sequencing, we analyzed the entire non-repetitive genomic sequence, including intronic and regulatory sequences, of 15 CRC susceptibility genes. In addition, tumor DNA from 28 sLS patients was analyzed for somatic MMR variants.

**Results:**

Of 1979 germline variants found in the leukocyte DNA of 34 sLS patients, one was a pathogenic variant (*MLH1* c.1667+1delG). Leukocyte DNA of 11 patients with *MLH1* hypermethylated tumors was negative for pathogenic germline variants in the tested CRC susceptibility genes and for germline *MLH1* hypermethylation. Somatic DNA analysis of 28 sLS tumors identified eight (29%) cases with two pathogenic somatic variants, one with a VUS predicted to pathogenic and LOH, and nine cases (32%) with one pathogenic somatic variant (n = 8) or one VUS predicted to be pathogenic (n = 1).

**Conclusions:**

This is the first study in sLS patients to include the entire genomic sequence of CRC susceptibility genes. An underlying somatic or germline MMR gene defect was identified in ten of 34 sLS patients (29%). In the remaining sLS patients, the underlying genetic defect explaining the MMRdeficiency in their tumors might be found outside the genomic regions harboring the MMR and other known CRC susceptibility genes.

## Introduction

Lynch Syndrome (LS) is the most common form of hereditary colorectal cancer (CRC) and is caused by heterozygous pathogenic germline variants in one of the mismatch repair (MMR) genes (*MLH1*, *MSH2*, *MSH6*, *PMS2*) [1]. In addition, a unique subgroup of LS patients carry deletions in the 3’ end of *EPCAM*, a gene immediately upstream of *MSH2* [2, 3].

More than 95% of LS-associated CRCs display microsatellite instability (MSI), the molecular hallmark of LS [4]. Immunohistochemical analysis (IHC) of the tumor for loss of MMR protein expression points to a possible causative gene, with the diagnosis of LS confirmed once a pathogenic germline variant is identified. Patients suspect for LS are selected for genetic testing on the basis of clinical characteristics (Amsterdam or Bethesda criteria) and/or molecular diagnostic testing of the LS-associated tumors (LSAT) [5, 6]. Opposed to familial colorectal cancer type X (FCCTX) families [7], who also fulfill Amsterdam criteria, the patients suspect for LS do show MSI and loss of MMR gene expression in the tumor.

LS patients have an increased risk of developing CRC, with estimates of lifetime risk ranging from 36% to 75% [8–11]. Women who carry pathogenic variants also face a high risk of endometrial cancer [12]. Several other cancer types, including small bowel, stomach, pancreas, ovary, renal pelvis, ureter, bladder, brain, liver, bile duct, gall bladder and skin occur frequently [11, 13–15]. Recent studies also indicate an increased risk for prostate and breast cancer [16–18]. To achieve adequate cancer prevention, early identification of individuals with LS is essential. Intensive surveillance by colonoscopy every 1–2 years, starting at age 20 to 25, is now recommended and is known to reduce CRC morbidity and mortality [19, 20]. Accurate and timely identification of LS patients is therefore crucial to providing the correct treatment [21].

A recent study estimated that, using current approaches, up to 60% of MMR-deficient colorectal cancer cases remain unexplained [21]. These patients are designated as ‘suspected Lynch Syndrome’ (sLS) [21], or also known as ‘Lynch-Like Syndrome’ [22], and failure to determine the underlying cause of disease has a major impact on the clinical management of these patients. In addition to germline variants, biallelic somatic variants may explain disease in up to 69% of MMR-deficient tumors that lack pathogenic germline variants or *MLH1* promoter hypermethylation [23–25].

MSI due to somatic hypermethylation of the promoter region of *MLH1* is also seen in up to 15% of sporadic CRC patients [26]. Sporadic *MLH1* methylated tumors commonly occur at a relatively advanced age and in absence of family history of CRC [27–29]. Patients with somatic *MLH1* promotor hypermethylated tumors rarely carry germline MMR variants, although exceptions have been published [30–32]. These studies indicate *MLH1* hypermethylation as a ‘second-hit’ mechanism already present in adenoma stage and demonstrate that *MLH1* hypermethylation does not exclude the presence of germline pathogenic MMR variants. *MLH1* hypermethylated tumors in young patients with a family history of CRC can also be due to germline *MLH1* hypermethylation. Though very rare, this phenomenon has been described before [33–41]. Inheritance of a constitutional epimutation has been shown in at least three unrelated families [42–44].

The aim of our study was to identify an underlying genetic basis in a cohort of 34 sLS patients and 11 patients with *MLH1* hypermethylated tumors and a clear family history for LS. In an effort to discover previously undetected germline variants, the entire genomic sequences of four MMR genes and eleven CRC susceptibility genes were analyzed. In addition, tumor DNA from 28 sLS tumors was analyzed for somatic variants in the MMR genes.

## Materials and Methods

### Study subjects

Between 1998 and 2011, a total of 45 patients were recruited from five academic centers in The Netherlands, including Leiden University Medical Centre (n = 20), Maastricht University Medical Centre (n = 11), Erasmus Medical Centre (n = 7), University Medical Centre Utrecht (n = 6) and VU University Medical Centre Amsterdam (n = 1). Demographic and clinical data and informed consent were obtained during the consultation. Forty-three patients fulfilled the revised Bethesda criteria [6].

All patients had been previously screened for germline variants in the MMR gene that showed loss of expression (as indicated by immunohistochemical analysis) by Sanger sequencing or denaturing gradient gel electrophoresis (DGGE), without identification of a pathogenic germline variant. Large deletions/duplications in the MMR genes were excluded by analysis with multiplex ligation-dependent probe amplification (MLPA, MRC Holland, Amsterdam), or in some cases, with Southern blot analysis.

Immunohistochemical analysis (IHC) and microsatellite instability testing were routinely performed at the request of a board-certified Clinical Genetic medical specialist. Because routine testing of all four MMR proteins only became available around 2004, tumors recruited before 2004 were not fully tested by MMR immunohistochemistry. Leukocyte and tumor DNAs were retrieved from the archives for the current study. Immunohistochemistry data was complete for 18 sLS patients (53%), for 10 cases only PMS2 immunohistochemistry was missing and the remaining 6 tumors had incomplete IHC results (see S1 Table). Ten *MLH1* hypermethylated tumors (8 colorectal-, 2 endometrium-) showed IHC loss of MLH1 and PMS2 (PMS2 was not tested in sLS-68—see S1 Table) and normal MSH2/MSH6 expression. The eleventh patient, sLS-81, showed loss of MLH1 expression (other MMR genes were not tested). All tumors except tumor sLS-48 (MSI not tested) displayed a microsatellite instable phenotype (high or low instability, see Table 1 and S1 Table).

**Table 1:** Clinicopathologic factors sLS- and *MLH1* hypermethylated cohort.

Clinicopathologic factor	no of patients (%)	
	sLS	*MLH1* hypermethylated
Number of patients	34	11
		
**Patient characteristics**		
Male	17 (50)	3 (27)
Female	17 (50)	8 (73)
Age, y	48,6	63,2
		
**Clinical characteristics**		
No Bethesda/Amsterdam II	1 (3)	1 (9)
Bethesda only	23 (68)	7 (64)
Amsterdam II	10 (29)	3 (27)
		
**Tumor**		
CRC	28 (82)	9 (82)
EC	5 (15)	2 (18)
Other	1 (3)	-
		
**Family History**		
FDR	28 (82)	11 (100)
NA	3 (9)	-
No	3 (9)	-
		
**MSI**		
MSI-High	25 (74)	9 (82)
MSI-Low	4 (12)	1 (9)
MSI-Stable	4 (12)	-
Unknown	1 (3)	1 (9)

Clinicopathologic factors of the 34 sLS- and 11 MLH1 hypermethylated patients. Patients presented with colorectal cancer (CRC), endometrial cancer (EC) or other LS-associated tumors (Other). Family history is defined as first degree relative with a LSAT (FDR), no family history of LS (No) or family history not available (NA).

Family history data showed that 82% of the sLS patients and 100% of patients in the *MLH1* hypermethylated cohort had a first-degree relative with a Lynch Syndrome-associated tumor (LSAT). Unfortunately, no DNA could be obtained from these affected family members. Among the sLS cohort, 28 patients presented with colorectal cancer (CRC) as their first LSAT, while 5 patients had endometrial cancer (EC) and 1 patient had a sebaceous gland cancer. In the *MLH1* hypermethylated cohort, 9 patients presented with CRC and 2 with EC. The mean age of diagnosis of the first LSAT was 48,6 years for the sLS group and 63,2 years for the *MLH1* hypermethylated group (See Table 1). Leukocyte DNA isolated from peripheral blood was available for all patients. The study was approved by the local medical ethical committee of the LUMC (P01-019E).

### Targeted genomic sequencing with next-generation sequencing

Targeted next-generation sequencing of leukocyte DNA was carried out using a custom designed set of SureSelect 120-mer target enrichment RNA oligonucleotides (baits) for in-solution hybrid selection (Agilent Technologies, Santa Clara, CA). Baits were designed against 15 CRC susceptibility genes, spanning the entire non-repetitive genomic region of the genes, including exons, introns, and UTRs, and 5 kb upstream and 3 kb downstream of the gene (see Table 2). The average coverage was > 95% for all coding regions, and 43% for overall coverage. Libraries were prepared according to the manufacturers’ protocols (NEBNext® and Illumina®, San Diego, California, USA). In brief, 2 μg of genomic DNA from each patient was fragmented to lengths of 300–500 bp using the Covaris S220 single tube sonicator (Life Technologies, Carlsbad, CA). Fragment ends were repaired and an A-tail was added to the 3’ end of the DNA fragments. Illumina® dual-barcoded adaptors (patient-specific) were ligated, and the adaptor-ligated DNA was then enriched by 10 cycles of PCR. PCR products derived from 4 to 5 patients were pooled in equimolar amounts and hybridized in solution to the custom baits. Captured targets were subsequently extracted and further enriched by 15 cycles of PCR. Paired-end sequencing of the PCR products was performed on the Illumina HiSeq® 2000.

**Table 2:** Custom-designed baits used for Sureselect target enrichment of 15 CRC susceptibility genes.

Chr	Genbank reference	Gene	Total target region (kb)	Chromosome		Total area covered (kb)	% of total target area	% repeated sequences
				Start	End			
1	NM_001128425.1	*MUTYH*	19,2	45791914	45811142	10,7	56%	33%
2	NM_002354.2	*EPCAM*	25,9	47591286	47617165	13,4	52%	55%
2	NM_000251.2	*MSH2*	88,1	47625262	47713360	26,1	30%	58%
2	NM_000179.2	*MSH6*	31,9	48005220	48037084	14,5	45%	42%
2	NM_000534.4	*PMS1*	101,5	190643810	190745354	48,4	48%	41%
3	NM_000249.3	*MLH1*	60,5	37034840	37095335	23,2	38%	52%
5	NM_002439.4	*MSH3*	230,3	79945293	80175633	80,6	35%	53%
5	NM_000038.5	*APC*	146,7	112038217	112184935	60,6	41%	47%
7	NM_000535.5	*PMS2*	36,7	6012370	6049037	9,8	27%	59%
10	NM_004329.2	*BMPR1a*	176,5	88511395	88687944	52,7	30%	58%
10	NM_000314.4	*PTEN*	103,3	89628194	89731531	41,8	40%	49%
14	NM_001040108.1	*MLH3*	43,8	75477466	75521235	21,3	49%	41%
16	NM_003502.3	*AXIN1*	73	334439	407464	39,1	54%	36%
17	NM_004655.3	*AXIN2*	118,5	63521684	63640183	65	55%	34%
18	NM_005359.5	*SMAD4*	62,8	48551582	48614409	31,7	50%	39%

Baits were designed against 15 CRC susceptibility genes. Target region spans the entire genomic region, including 5 kb upstream and 3 kb downstream of the gene. Repeated sequences are not covered by custom-designed baits.

### Data analysis

Illumina HiSeq® 2000 sequences were exported as FASTQ files and separated using the barcodes. The sequence data was checked for quality using the quality control tool for high throughput sequence data, FastQC (http://www.bioinformatics.babraham.ac.uk/projects/fastqc/). Alignment of the Illumina sequences to the human reference genome (hg19, NCBI build GRCh37) was performed using the Burrows-Wheeler aligner (BWA) (http://bio-bwa.sourceforge.net) and sequence duplicates were marked with Picard (*http*:*//picard*.*sourceforge*.*net/*). Variant calling on the resulting BAM files was performed by VarScan (*http*:*//varscan*.*sourceforge*.*net/*) using the following settings: minimal coverage of 8, minimal reads of 2, minimal variant frequency of 0.2 and a minimal average quality of 20. Variants were functionally annotated using ANNOVAR [45].

### Variant filtering and classification

The full dataset was filtered by targeted region and variant frequency. Variants with a minor allele frequency (MAF) of >0.05, as reported in the NCBI dbSNP database version 142 (http://www.ncbi.nlm.nih.gov/projects/SNP/) were excluded from further analysis. Because analysis of *PMS2* variants is difficult due to interference by pseudogenes, variants located in one of the duplicated regions were excluded from further analysis.

#### Splice variants

All remaining sequence variants with a MAF<0.05 were analyzed with Alamut software version 2.0 (Interactive Biosoftware, Roven, France), a software package that includes the splice site prediction algorithms SpliceSiteFinder, MaxEntScan (*http*:*//genes*.*mit*.*edu/burgelab/maxent/Xmaxentscan_scoreseq*.*html*), NNSPLICE (*http*:*//www*.*fruitfly*.*org/seq_tools/splice*.*html*) and Human Splicing Finder (*http*:*//www*.*umd*.*be/HSF/*). Variants can affect splicing by altering the canonical splice site sequence, by creation of new splice sites, activation of cryptic splice sites or by altering splice regulatory elements (SREs) [46]. In addition, branch point sequences and polypyrimidine tracts were investigated for possible variants. As a branch point is usually located 18 to 50 nt upstream of the splice acceptor site, all variants within 100 nt of the splice acceptor sites of *MLH1*, *MSH2*, *MSH6* or *PMS2* were visually inspected in Alamut [47].

#### Missense prediction

All missense variants were filtered based on the predictions of *in silico* protein prediction software packages including Align GVGD, SIFT (*http*:*//sift*.*jcvi*.*org/*), PolyPhen-2 (*http*:*//genetics*.*bwh*.*harvard*.*edu/pph2/*), MutationTaster (*http*:*//www*.*mutationtaster*.*org/*) and MutationAssessor[48].

#### Promoter variants

Using the UCSC Genome browser, the putative promoter regions of *MLH1*, *MSH2*, *MSH6* and *PMS2* (as indicated by the histone mark H3K4me3 that is generally found near promoters) were analyzed for variants.

#### Variant classification

The Leiden Open Variation Database (LOVD, http://www.lovd.nl/3.0/home) and ClinVar [49] were consulted to identify previously described and classified variants. Variants that were not described in above-mentioned databases were classified according to the five-tiered InSiGHT scheme: benign (class 1), likely benign (class 2), variant of unknown significance (class 3), likely pathogenic (class 4), and certainly pathogenic (class 5) [50].

#### Validation

All (likely) pathogenic- or splice variants were visually inspected in the Integrative Genomics Viewer (IGV, *https*:*//www*.*broadinstitute*.*org/igv/home*) and confirmed with Sanger Sequencing.

Germline variants found in this study have been submitted to the appropriate LOVD database, available at http://www.lovd.nl/3.0/home.

### *MLH1* promoter hypermethylation

Methylation of the *MLH1* promoter region was analyzed using methylation specific PCR (MSP), with previously described primers [51]. Bisulfite conversion of tumor DNA was carried out using the EZ DNA methylation Gold kit (Zymo Research, Orange, US), following the manufacturers’ standard protocol. Bisulfite-converted DNA was amplified using specific methylated and unmethylated primers in a PCR reaction, following a LUMC diagnostics protocol [33].

### Functional RNA analysis

To determine the effect on splicing of one splice site variant (*MLH1* c.1667+1delG), patient RNA was analyzed for aberrant splicing. RNA was isolated from short-term cultured peripheral blood lymphocytes, and analyzed with and without inhibition of nonsense-mediated decay [46]. In addition, a minigene splicing assay was performed to confirm the splicing patterns seen in the RNA of the patient as described by van der Klift et al [46].

### Somatic variant screening

DNA from 28 sLS tumors, isolated from formalin-fixed paraffin-embedded tissue blocks, was screened for variants in the coding regions of *MLH1*, *MSH2*, *MSH6* and *PMS2* with the Ion PGM™ (Life Technologies, Carlsbad, CA). Next-generation sequencing was carried out according to the Ion PGM™ protocol, with supplier’s materials. Primers were obtained from Life Technologies. The complete panel consisted of 162 amplicons, covering 98%, 90%, 98% and 75% of *MLH1*, *MSH2*, *MSH6* and *PMS2*, respectively.

Raw data analysis, alignments, and variant calling were carried out using the default parameters in Torrent Suite v4.0. The Variant Caller Parameter Setting was set on ‘Somatic–PGM–Low Stringency’. Variants were functionally annotated using ANNOVAR [45]. Variants were visually inspected with IGV and (likely) pathogenic variants were confirmed with Sanger sequencing. The annotated dataset from the somatic screening was filtered in the same manner as the germline targeted next-generation sequencing dataset. For assessment of pathogenicity, the catalogue of somatic mutations in cancer (COSMIC, http://cancer.sanger.ac.uk/cosmic) was used additionally. Loss of heterozygosity (LOH) was determined as previously described [52]. Somatic data on 20 sLS tumors and 3 *MLH1* hypermethylated tumors (see S1 Table) has been described previously [52]. These twenty-three patients were also tested for variants in the *POLE/POLD1* exonuclease domain (EDM). Patient sLS-07 and sLS-09 were found to carry *POLE*-EDM variants, previously described to be pathogenic (respectively *POLE* c.2131 G>T and *POLE* c.857 C>G) [52, 53].

## Results

### Germline targeted next-generation sequencing

#### Coding variants

Targeted next-generation genomic sequencing of 15 CRC susceptibility genes was performed in leukocyte DNA of 34 unrelated sLS patients and 11 patients with *MLH1* hypermethylated tumors. The average coverage was 101x. In total, 1979 nucleotide variants were detected within the targeted region with a MAF < 0.05. All 15 genes were first analyzed for coding variants. After filtering, 52 coding variants remained, of which 16 were synonymous, 33 were missense and 3 were small (in-frame) insertions or duplications. All in-frame insertions/duplications occurred within a stretch of Ala-repeats in exon 1 of *MSH3* and were present in multiple patients and were classified as variants of unknown (clinical) significance (VUS). Eight of the 33 missense variants were found in the coding sequences of *MLH1*, *MSH2*, *MSH6* or *PMS2* and were described in the LOVD database as (likely) benign (class 1 or 2), except *MLH1* c.277A>G, which was classified as VUS (class 3). Of the remaining 25 missense variants, 20 were predicted to be benign by at least four out of five protein prediction programs. One of the remaining five variants, *EPCAM* c.50C>A was predicted to be pathogenic by two out of five prediction programs but was described to be benign [49]. The final 4 variants were found in *AXIN1*, *AXIN2*, *MSH3* and *MUTYH* and were classified as variants of uncertain significance (VUS), or as pathogenic (n = 1; *MUTYH* c.1187G>A) (see Table 3).

**Table 3:** Patients with germline coding VUS or germline pathogenic variants.

Patient	Tumor tested	IHC negative staining	MSI	Other tumors	Gene	Variant	Protein	Class
sLS-22	CRC54	MLH1^1^	H	-	*MUTYH*	c.1187G>A	p.(G396D)	5
					*MLH1*	c.277A>G	p.(S93G)	3
sLS-44	CRC41	MSH2/MSH6	H	-	*AXIN2*	c.1168A>G	p.(S390G)	3
sLS-56	CRC64	MSH2/MSH6	H	CRC64	*AXIN1*	c.2566G>A	p.(G856S)	3
sLS-72	CRC73	MSH2/MSH6	H	Br60, EC68	*MUTYH*	c.1187G>A	p.(G396D)	5
sLS-88	CRC51	MLH1/PMS2	H	Pr64	*MSH3*	c.982C>T	p.(R328W)	3
sLS-117	CRC20	PMS2	NP	-	*MLH1*	c.1667+1delG	p.(S556ins29)	5

^1^staining of MSH6 and PMS2 was not performed. Tumor tested represents tumor type, followed by the age of onset. Patients presented with colorectal- (CRC), endometrial cancer (EC), breast cancer (Br) and/or Prostate cancer (Pr). MSI-status is defined as MSI-High (H) or not performed (NP). Classification of class 3 (VUS) and class 5 (pathogenic) is based on in silico protein predictions, as well as the LOVD Database. All variants were found in sLS patients.

#### Splice variants

For three variants the splice prediction algorithms predicted deviating splicing efficiencies compared to the wildtype sequences. An *MLH1* variant, in patient sLS-117 (see Table 3), was predicted to abolish the consensus splice site sequence (c.1667+1delG). Functional analysis of patient RNA revealed a mutant MLH1 transcript 87 nucleotides longer than the expected wild type transcript [46]. The 87 nt sequence corresponded to the intron sequence downstream of the splice variant, indicating activation of a cryptic donor splice site 88 nucleotides downstream of the canonical splice site. Translation of the aberrant mRNA leads to the in-frame incorporation of 29 amino acids in the protein-interacting domain of the MLH1 protein. The other variants predicted to affect splicing, a synonymous *APC* c.1959G>A change and the *MUTYH* c.1187G>A variant described above, only slightly lower the splicing efficiency according to prediction software. The *APC* variant is described in the LOVD database as having ‘no known pathogenicity’.

In addition, branch point sequences and polypyrimidine tracts were investigated for possible variants with branch site prediction software SpliceSiteFinder. None of the variants found were predicted to change the existing consensus sequence or to create new branch points.

#### Promoter variants

Of the 22 promoter variants, 8 were known polymorphisms. The remaining 14 variants were present in single patients of which three were actually present in the specific MMR gene that showed loss of protein expression in the tumor: *MLH1* c.-1019A>C, *MLH1* c.116+730C>T and *MSH2* c.211+550G>C. These variants have not been described before, and functional significance of these variants is unknown according to the INSIGHT classification [50].

#### Germline MLH1 methylation

Leukocyte DNA of patients with *MLH1* hypermethylated tumors were also investigated for possible germline methylation. No evidence of germline methylation was found in any of the patients

### Somatic variant screening

Tumor DNA from 29 of the 34 sLS tumors was available for somatic DNA analysis. Patient sLS-117 was excluded from somatic variant screening due to the detection of a pathogenic germline *MLH1* variant (*MLH1* c.1667+1delG). Tumor and normal DNAs from the remaining 28 patients were sequenced for somatic MMR variants.

In total, two pathogenic somatic events were detected in eight tumors (29%), including either two variants (n = 3) or one variant together with LOH (n = 5) (see Table 4 and S1 Table). One tumor was found to carry a VUS (predicted to be pathogenic) together with LOH. Nine tumors (32%) revealed one pathogenic somatic variant (n = 8), or VUS predicted to be pathogenic (n = 1), while no (likely) pathogenic somatic variants were found in seven of the tumors (25%) (see Table 4). Three tumors (11%) could not be analyzed due to poor tumor DNA quality. Seventeen out of the twenty-two somatic MMR variants were nonsense or frameshift variants and were classified as pathogenic (class 5). Of the remaining five somatic variants, two (*MLH1* c.790+1 G>A and *MLH1* c.2059C>T) were previously described to be pathogenic in the LOVD database; two (*MSH6* c.2876 G>A, and *MSH2* c.1166G>A) were not previously described and were predicted to have a deleterious effect on function by at least four out of five protein prediction programs (See Table 4) and one was an in-frame deletion of three nucleotides (*MSH6* c.3974_3976delAGA), which was classified as having an uncertain effect on function (VUS, class 3).

**Table 4:** Patients screened for somatic variants.

Patient	Tumor tested	Cohort	MSI	Family History	Gene	Variant	Amino acid alteration	%	Class	Functional annotation
**Two somatic variants**										
sLS-06	CRC47	MSH2/MSH6	H	FDR	*MSH2*	c.1600_1601delCG	p.(R534*)	19%	5	Nonsense variant
					*MSH2*	c.2131 C>T	p.(R711*)	20%	5	Nonsense variant
sLS-07	CRC39	MSH6	S	FDR	*MSH6*	c.2876G>A	p.(R959H)	14%	3	VUS, 4/5 programs predict pathogenic
					*MSH6*	LOH				
sLS-22^a^	CRC54	MLH1/PMS2	H	FDR	*MLH1*	c.281delT	p.(S95Lfs*13)	93%	5	Frameshift variant
					*MLH1*	LOH				
sLS-38^a^	CRC30	MSH2/MSH6	H	FDR	*MSH2*	c.1140delA	p.(L380Ffs*32)	82%	5	Frameshift variant
					*MSH2*	LOH				
sLS-79^b^	EC57	MSH2/MSH6	H	FDR	*MSH2*	c.1600delC	p.(R534Vfs*9)	20%	5	Frameshift variant
					*MSH2*	c.2001delT	p.(T668Lfs*17)	20%	5	Frameshift variant
sLS-92	CRC45	MLH1/PMS2	H	FDR	*MLH1*	c.790+1 G>A	p.(E227_S295del)	78%	5	Pathogenic (LOVD database)
					*MLH1*	LOH				
sLS-102^a^	CRC62	MLH1/PMS2	H	FDR	*MLH1*	c.869dupC	p.(F291Ifs*16)	53%	5	Frameshift variant
					*MLH1*	LOH				
sLS-104	SB47	MSH2/MSH6	H	No	*MSH2*	c.271delG	p.(D91Ifs*83)	92%	5	Frameshift variant
					*MSH2*	LOH				
sLS-111	EC58	MSH2/MSH6	H	FDR	*MSH2*	c.687delA	p.(I229Mfs*10)	44%	5	Frameshift variant
					*MSH2*	c.773 T>A	p.(L258*)	41%	5	Nonsense variant
**One somatic variant**										
sLS-09	CRC42	MSH6	L	FDR	*MSH2*	c.1166G>A	p.(R389Q)	38%	3	VUS, 4/5 programs predict pathogenic
					*MSH6*	c.2539G>T	p.(E847*)	36%	5	Nonsense variant
sLS-55	EC47	MSH2/MSH6	H	FDR	*MSH6*	c.3971delAGA	p.(L1325del)	24%	3	VUS, in-frame deletion
					*MSH2*	LOH				
sLS-56	CRC64	MSH2/MSH6	H	NA	*MSH2*	c.1710T>A	p.(Y570*)	19%	5	Nonsense variant
sLS-58	CRC39	MLH1/PMS2	L	FDR	*MLH1*	c.790+1 G>A	p.(E227_S295del)	28%	5	Pathogenic (LOVD database)
sLS-64	CRC48	MLH1/PMS2	H	FDR	*MLH1*	c.2059C>T	p.(R687W)	28%	5	Pathogenic (LOVD database)
sLS-72	CRC73	MSH2/MSH6	H	No	*MSH2*	c.1576dupA	p.(T526Nfs*3)	29%	5	Frameshift variant
sLS-77	CRC45	MSH2/MSH6	H	No	*MSH2*	c.2470C>T	p.(Q824*)	38%	5	Nonsense variant
sLS-101	EC55		H	FDR	*PMS2*	c.1687C>T	p.(R563*)	30%	5	Nonsense variant
sLS-127^a^	CRC45	MSH2/MSH6	H	NA	*MSH2*	c.2527delT	p.(C843Vfs*49)	37%	5	Frameshift variant
**No somatic variants**										
sLS-17	CRC39	MSH6	S	FDR	-	-	-	-		
sLS-20	CRC55	MSH2/MSH6	S	FDR	-	-	-	-		
sLS-43	CRC74	MSH2/MSH6	H	FDR	-	-	-	-		
sLS-44	CRC41	MSH2/MSH6	H	FDR	-	-	-	-		
sLS-62	CRC35	MSH2/MSH6	L	FDR	-	-	-	-		
sLS-82	CRC69	MLH1/PMS2	H	FDR	-	-	-	-		
sLS-120	CRC57	MLH1/PMS2	S	FDR	-	-	-	-		

^a^ Somatic screening was performed by the Erasmus MC, Rotterdam.

^b^ Somatic screening was performed by the Radboud UMC, Nijmegen.

Patients presented with colorectal cancer (CRC), endometrium cancer (EC) or sebaceous gland cancer (SB). Tumor tested shows tumor type followed by patients age of onset. Cohort gives an indication of IHC results. Detailed IHC results are shown in S1 Table. MSI-status is defined as MSI-High (H), MSI-Low (L) or MSS (S). Family history is defined as first-degree relatives with LSAT (FDR), no LSAT within the family (No), or family history not available (NA). Stopcodons are indicated with an asterisk (*). % shows the percentage of variant reads. All variants are validated with Sanger sequencing. Two variants are predicted to be pathogenic by at least 4 out of 5 of the following programs: Align GVGD, SIFT, MutationTaster, Polyphen and MutationAssessor.

Patient sLS-22 was previously found to carry a germline *MLH1* VUS (*MLH1* c.277 A>G), and analysis of the tumor DNA revealed an somatic *MLH1* frameshift variant located nearby the germline variant (*MLH1* c.281delT). NGS analysis showed that both variants were located on the same allele. Moreover, the tumor DNA displayed LOH with retention of both variants.

### MMR mosaicism

To investigate the possibility of mosaic MMR variants, all cases in which a somatic MMR variant was identified were tested for mosaicism in the corresponding leukocyte DNA. The average coverage of the leukocyte DNA samples was more than a thousand reads per amplicon and no mosaic variant was detected.

## Discussion

In this study we carried out an extensive sequencing analysis of the genomic regions of the four MMR and 11 other CRC susceptibility genes, including *MUTYH*, *EPCAM* and *MSH3*. We anticipated that this type of broad analysis, well beyond the boundaries of conventional mutation screening, would identify variants previously missed by standard techniques or would identify variants in genes other than the previously diagnostically tested MMR genes. As our patient cohort consisted mainly of cases with a first-degree relative with a LS-associated tumor, cancer susceptibility due to an underlying germline defect in these families seemed the most plausible explanation.

The approach used, Whole Gene Capture, yielded an average sequence depth up to 5-fold greater than whole exome sequencing, with sufficient depth to allow detection of mosaic and *de novo* variants. In total, 1979 initial variants were detected. Many variants were classified as of uncertain significance and follow-up studies might reveal novel functional effects. After filtering by function and predicted pathogenicity, two likely pathogenic variants remained. An *MLH1* splice site variant, c.1667+1delG, was found in patient sLS-117, who was diagnosed with CRC at age 20. Patient sLS-117 presented with solitary PMS2 protein deficiency in the tumor and only *PMS2* had been previously screened with conventional mutation screening. IHC showed solitary PMS2 loss of expression, since the *MLH1* frameshift variant leads to a 29 amino acid insertion in the protein-protein interacting domain, resulting in an MLH1 transcript which is unable to bind PMS2. Analysis of family members demonstrated the variant in the patient’s affected mother (CRC at age 44), whereas the patient’s unaffected daughter tested negative for the variant.

The second pathogenic variant, *MUTYH* p.Gly396Asp, was present in a heterozygous state in two patients (patient sLS-22 and sLS-72, see Table 3). Monoallelic variants in *MUTYH* are present in 2% of the general population and are not found at increased frequencies in sLS patients [54, 55]. The role of monoallelic *MUTYH* variants is still under debate, and while some studies have indicated an increased cancer risk for carriers of a single *MUTYH* variant, the p.Gly396Asp variant alone is unlikely to be the explanation for the MSI-H and/or IHC status of the tumors in our patients [56, 57]. Moreover, both patients were found to have (likely) pathogenic somatic MMR variants (S1 Table) explaining the MMR-deficient phenotype.

In addition to the 34 sLS patients, eleven colorectal cancer patients with somatic *MLH1* hypermethylation and a family history suspected of LS were analyzed for possible underlying germline defects. *MLH1* promoter methylation in Lynch Syndrome patients has been described before, either co-occuring with a pathogenic germline *MSH6* variant in a patient with a urothelial carcinoma at age 70 [30], in a patient with a pathogenic germline *MLH1* variant in a CRC at the age of 59 [31] or with pathogenic germline *MSH6* variant in a patient with multiple primary cancers, from the age of 56 [32]. Another study describes *MLH1* hypermethylation in three LS-tumors, hypothesizing methylation is the second hit inactivating the wildtype allele [58]. These studies indicate that *MLH1* hypermethylation does not always exclude a diagnosis of LS. In our study we have not find support for above findings.

Moreover, three families with germline *MLH1* hypermethylation in multiple affected family members have been reported [42–44], indicating epigenetic inheritance of constitutional epimutations with a risk of transgenerational inheritance. All eleven patients with *MLH1* hypermethylated tumors in our cohort were tested for germline methylation, but no germline methylation was found.

Although this intensive study enabled the detection of variants within the intronic regions, UTRs and regions up- and downstream of the target genes, some limitations have to be considered. While the average coverage of the coding regions is over 95%, the overall average coverage is 43% (See Table 2). The lower overall coverage is due to the method used in which no baits were designed for the repetitive sequences such as the Alu- and Line-repeats within the introns. Therefore, missed intronic variants in these regions cannot be excluded. Moreover, we cannot exclude the possibility of large genomic rearrangements within the genes tested, which is a limitation of the method used in this study.

Screening of tumor DNA from 28 sLS patients for somatic variants revealed almost a third with two somatic variants (n = 3) or a combination of a somatic variant and LOH (n = 6). The frequency of biallelic inactivation in our cohort is lower than previously described [23–25], and might be due to differences in patient selection in the different study cohorts. While previous studies screened sLS patients irrespective of family history, the majority of patients in the present cohort had first-degree relatives with LS-associated tumors (see S1 Table) and eight families even fulfilled the Amsterdam II criteria. However, while biallelic somatic events may explain the MMR deficiency of the tumor of the index patient, they cannot explain a family history of CRC. Ideally, a second affected family member in these families should be tested to see whether these patients can also be explained by somatic MMR inactivation. Unfortunately, no DNA could be obtained from affected family members. An underlying pathogenic germline gene variant outside these 15 CRC susceptibility genes cannot be excluded in these families.

Besides somatic MMR variants, two sLS patients (sLS-07 and sLS-09) were recently found to carry somatic hotspot *POLE*-EDM variants (see S1 Table) [52]. As *POLE/POLD1*-EDM pathogenic variants give rise to ultramutated tumors, the somatic MMR variants apparently represent a second hit. Screening for germline or somatic *POLE/POLD1*–EDM variants, but also for variants in other genes recently described to be mutated in sLS CRCs such as *BRCA1*, *BRCA2*, *ATM* and *CHEK2*, may explain some of these sLS patients [59, 60]

In conclusion, sequencing of the entire genomic region of 15 CRC susceptibility genes in 34 unrelated sLS patients and 11 patients with *MLH1* hypermethylated tumors, together with assessment of somatic variants, provides a broad impression of possible genetic causes of tumor formation in MSI-H and/or MMR-deficient tumors. No likely pathogenic MMR gene variants or germline *MLH1* hypermethylation were found that explained the familial aggregation of cancer susceptibility in any of the families with *MLH1* hypermethylated tumors. With the MMR deficiency of around one-third of the 34 sLS tumors now explained, MMR deficiency in two-thirds of sLS tumors remains genetically unaccounted for. A logical next step is whole exome sequencing (WES) or whole genome sequencing (WGS) to further elucidate the causative genetic defect(s) in the remaining patients.

## Supporting Information

S1 Table Overview of germline and somatic variants found in 34 sLS patients and 11 patients with an *MLH1* hypermethylated tumor. (XLSX)
